# Common mental disorders among adult members of ‘left-behind’ international migrant worker families in Sri Lanka

**DOI:** 10.1186/s12889-015-1632-6

**Published:** 2015-03-28

**Authors:** Chesmal Siriwardhana, Kolitha Wickramage, Sisira Siribaddana, Puwalani Vidanapathirana, Buddhini Jayasekara, Sulochana Weerawarna, Gayani Pannala, Anushka Adikari, Kaushalya Jayaweera, Sharika Pieris, Athula Sumathipala

**Affiliations:** Faculty of Medical Science, Anglia Ruskin University, Chelmsford, CM1 1SQ UK; Institute for Research & Development, Colombo, Sri Lanka; International Organization for Migration, Colombo, Sri Lanka; Department of Medicine, Faculty of Medicine & Allied Sciences, Rajarata University of Sri Lanka, Anuradhapura, Sri Lanka; Department of cardiothoracic vascular surgery, National University Hospital, Singapore, Singapore; Ministry of Health, Colombo, Sri Lanka; Research Institute for Primary Care and Health Services, School for Primary Care Research, Faculty of Health, Keele University, Newcastle, UK

**Keywords:** International migrant workers, Economic migration, Migration health, Mental health, Left-behind families, Health policy, Labour sending country, Developing world, Sri Lanka

## Abstract

**Background:**

Nearly one-in-ten Sri Lankans are employed abroad as International migrant workers (IMW). Very little is known about the mental health of adult members in families left-behind. This study aimed to explore the impact of economic migration on mental health (common mental disorders) of left-behind families in Sri Lanka.

**Methods:**

A cross-sectional survey using multistage sampling was conducted in six districts (representing 62% of outbound IMW population) of Sri Lanka. Spouses and non-spouse caregivers (those providing substantial care for children) from families of economic migrants were recruited. Adult mental health was measured using the Patient Health Questionnaire. Demographic, socio-economic, migration-specific and health utilization information were gathered.

**Results:**

A total of 410 IMW families were recruited (response rate: 95.1%). Both spouse and a non-spouse caregiver were recruited for 55 families with a total of 277 spouses and 188 caregivers included. Poor general health, current diagnosed illness and healthcare visit frequency was higher in the non-spouse caregiver group. Overall prevalence of common mental disorder (CMD; Depression, somatoform disorder, anxiety) was 20.7% (95%CI 16.9-24.3) with 14.4% (95%CI 10.3-18.6) among spouses and 29.8% (95%CI 23.2-36.4) among non-spouse caregivers. Prevalence of depression (25.5%; 95%CI 19.2-31.8) and somatoform disorder 11.7% (95%CI 7.0-16.3) was higher in non-spouse caregiver group. When adjusted for age and gender, non-returning IMW in family, primary education and low in-bound remittance frequency was associated with CMD for spouses while no education, poor general health and increased healthcare visits was significantly associated in the non-spouse caregiver group.

**Conclusions:**

To our knowledge, this is one of the first studies to explore specific mental health outcomes among adult left-behind family members of IMW through standardized diagnostic instruments in Sri Lanka and in South Asian region. Negative impact of economic migration is highlighted by the considerably high prevalence of CMD among adults in left-behind families. A policy framework that enables health protection whilst promoting migration for development remains a key challenge for labour-sending nations.

## Background

Migration for economic reasons has become an important geo-political phenomenon of the modern era [1,2]. The growing aspirations of economic migrants are driven by the market demands of rapidly developing economies in the world. International migrant workers (IMW) from Sri Lanka have grown ten-fold during the past decade and around 17% of Sri Lanka’s total labour force is currently working outside its borders [3]. Middle Eastern region is the main destination for Sri Lankan IMWs, and an average of 720 registered migrant workers departed Sri Lanka each day for foreign employment in 2011 [4]. This figure may be considerably higher if volumes of irregular and unregistered migrant workers can be estimated. Majority of Sri Lankans are employed abroad as ‘domestic maids or labourers’ [4]. Overall contribution from the economic migrant remittances to the Sri Lankan economy equals up to 8% of the total GDP [3,5].

Despite the monetary benefits to migrants, their families and to the country, evidence is emerging about numerous unfavorable effects of economic migration, including adverse health outcomes for IMWs and their left-behind families [6,7]. Although several studies have provided insights into the social, legal and economic impacts of economic migration in Sri Lanka, empirical evidence about the true scale of nation-wide health impact of economic migration is scarce [8-11]. A review of literature found three published studies from Sri Lanka which examined health status of left-behind children of migrant households [12-14]. There is no existing evidence of studies exploring health status of left-behind spouses or non-spouse caregivers, who has a role in providing care for children and other members of left-behind families. Although sparse, there is some evidence that migration of adult children for economic reasons can negatively affect both mental and physical health of aging parents [15]. In most extended and traditional family units in developing countries, the aging parents of migrant workers may be additionally tasked with caring for the grandchildren of the left-behind family, precipitating increased health problems [15]. Other studies have shown that spouses of out-migrants have increased physical and mental health problems, including depression [16]. Some studies on the impact of migration among left-behind parents of emigrants moving to developed countries have shown mixed results. Some studies reported that the adult left-behind family members have better living standards and does not have any negative effects on their health while others reported that parents of migrants have worse self-reported health [17-19].

In the global context, despite the political discourse on migration becoming an important issue in the global development agenda, the public health implications for migrants and their families have received little attention [20-22]. A PLoS medicine series on *Migration & Health* in 2011 prompted public health attention and called for an evidence-based research agenda on health of migrants [23]. The health impact of economic out-migration and families left-behind is especially salient for majority of labour-sending countries including Sri Lanka, which are mostly low and middle income countries (LMIC) without adequate health system resources to counter the interlinked public health issues [23,24]. Demographic and epidemiological shifts combined with international migration affect family structures, health and long-term care provision, labour force participation, retirement and financial security [25]. There remains a scarcity of studies on this topic despite an ever-growing international labour migration market.

The study described here aimed to explore the impact of economic migration on mental health of adult left-behind family members in Sri Lanka by establishing the prevalence of common mental disorders (CMD; depression, anxiety, somatoform disorder). It also aimed to explore associations between CMD and demographic, economic, social, migration-related and general health-related factors using standardized and culturally validated instruments.

## Methods

### Study setting, design and participants

A national study was conducted by the Institute for Research & Development in partnership with the International Organization of Migration and the Ministry of Health as part of the ‘National Migration Health Research’ agenda. It was recommended by the Government of Sri Lanka’s ‘Inter-Ministerial Taskforce on Migration Health’, and sought to contribute to an evidence-based ‘National Migration Health Policy’ formulation process. This larger commissioned study on ‘left-behind families’ contained both quantitative and qualitative components and included both adult and child members. The qualitative and child population findings are presented elsewhere [8,26].

This manuscript presents findings from the cross-sectional survey component conducted among adult family members in six districts (Colombo, Gampaha, Kurunegala, Kandy, Kalutara and Puttalam) with the highest number of outbound departures for foreign employment in Sri Lanka, representing 62% of the total migrant worker population [4,5]. The study included the family members of migrant workers (employed abroad for at least six months prior to recruitment), residing in one of the selected six districts. A migrant family was defined as a family unit where either one or both spouses have departed for employment abroad as an IMW, with their own or adopted child/children under 18 years of age, and residing at an address for at least a six month period at the time of data collection. The six month period was considered as a minimal threshold for continuity regarding employment abroad or residence at a given address. Left-behind family members recruited for the study included the spouse of the IMW and/or a non-spouse caregiver (defined for the study purposes as a person living in the migrant family household, not a parent of the child/children in the family but responsible for providing a significant amount of care for them on a daily basis). Non-spouse caregiver was further assessed for activities related to basic care of children such as; arranging schedules, preparing or ensuring meals, assisting in educational, social and health needs (including play), and providing guardianship and representation to authorities.

### Sampling and data collection

A multi-stage random sampling method was used to select left-behind families. In Sri Lanka, the smallest administrative units are called Grama Niladhari Divisions (GND)/ village units. Larger Divisional Secretariat Divisions (DSD) are made up from multiple GNDs. Several DSDs make up a district and Sri Lanka is divided into a total of 25 administrative districts. A total of 41 GNDs were randomly selected (1 GND from a DSD) from the six districts with the highest percentage of migrant worker representations. A probability proportionate to size sampling frame was adopted to capture the different rates of out-migration within the six districts.

In each selected GND, a list of all migrant families was compiled using information obtained through Grama Niladhari (GND/village administrator), Public Health Midwife (PHM), and Samurdhi Niyamaka (GND/village welfare worker). Lists were cross-checked with each other for accuracy and a final, verified list was prepared. Subsequently, using these finalized versions, ten migrant worker families were randomly selected from each GND. A total of 410 families were recruited for the study in accordance with the sampling calculations [27]. To mitigate limitations in sampling such as inaccurately maintained village registries, or in situations where selected families were not available, a single new GND or a family was randomly selected as the substitute. In migrant families where a spouse and non-spouse caregiver were available, both were approached for consent and subsequently recruited if consented. If there was more than one non-spouse caregiver available, the one who was mostly involved in the provision of care was approached.

Data collection was supervised and managed by two dedicated project coordinators and a statistician. Field work was conducted by a team of 22 trained field research assistants under the supervision and guidance from a team of experts consisting of a psychiatrist, physician, epidemiologist and two public health specialists. Interviews were administered by the research assistants using instruments described in the section below. Participant responses were recorded on the printed questionnaire booklet by the research assistants. In instances where participants faced difficulties in understanding questions asked, the research assistants were trained to explain the meaning of the question in lay language to the participants. Double data entry was conducted using the Statistical Package for Social Sciences (SPSS version 17) [28]. Ethical approval was granted by the Ethical Review Committee of Faculty of Medicine, University of Colombo. Informed written consent was obtained from all participants.

### Measurements

Basic social, economic, environmental and demographic indicators were captured. Variables included gender, ethnicity, family size, employment type, educational status, home ownership status, household setting/conditions, household goods, income and debt. Additional variables directly related to migration such as IMW return history, frequency of remittances from the IMW and type of employment of the IMW were measured. The categories of migrant worker employment were classified according to Sri Lanka Bureau of Foreign Employment definitions [4].

CMD (depression, anxiety and somatoform disorder) prevalence was measured using the Patient Health Questionnaire (PHQ). It is a scale derived from Primary Care Evaluation of Mental Disorders (PRIME‐MD), with demonstrated diagnostic performance (sensitivity - 83%; specificity - 88%) for the diagnosis of most CMD (somatoform, depressive, anxiety, eating and alcohol disorders) in primary health care according to Diagnostic and Statistical Manual-IV (DSM-IV) criteria [29,30]. However, it has also been used for estimating CMD prevalence in research populations, and has been translated, validated and applied widely in cross-cultural research [30-32]. A computer algorithm is used to generate ‘positive’ or ‘negative’ outcomes for each constituent disorder category in the PHQ [33]. A score between 10-15 is considered as clinically significant severity level with the upper limit requiring possible treatment [33].

The PHQ was used in this study as it has been previously validated using nominal techniques [34] and utilised in Sri Lanka for a number of epidemiological studies (National Mental Health Survey with a sample of 6000 participants, Colombo Twin and Singleton Study with a sample size of 6000, a study measuring CMD among displaced populations with a 450 sample) [35-37]. General health status, presence of current illness (clinically significant, diagnosed chronic conditions; e.g. Diabetes) and the number of visits to healthcare providers were ascertained from participants by using an adapted version of Client Service Receipt Inventory (CSRI) [38,39].

### Data analysis

Statistical analysis was conducted using STATA version 11 [40]. The data were weighted to account for the cluster sampling design and population size variations. Missing data were accounted for by using complete case analysis, which is default in STATA. Descriptive analyses were carried out to describe the sample characteristics and CMD prevalence. For analytical purposes, positive diagnoses for CMD constituent disorders (depression, anxiety, somatoform disorder) were grouped together to form the any CMD variable. A correlation matrix was generated using all variables to guide the analysis. Univariable logistic regression analyses were carried out to explore associations between CMD, socio-demographic, economic, migration-related and health-related variables. Multivariable logistic regression analyses for associations between CMD and the above mentioned variables were adjusted for age and gender.

## Results

Tables 1 and 2 summarize key demographic, economic, migration-related and health-related characteristics. A total of 410 migrant worker families were recruited for the study. Within those, only spouses were recruited from 222 families and only non-spouse caregivers from 133 families. Both a spouse and a non-spouse caregiver were recruited from 55 families. In total, 277 migrant worker spouses and 188 non-spouse care givers were recruited with a total sample of 465 individuals. Mean ages (SD) of the spouse and non-spouse caregiver groups were 37.8 (0.46) and 54.1(0.86) respectively. Non-spouse caregiver group was predominantly female (95.7%), with approximately a third over 60 years of age (29.3%). Forty five percent of recruited migrant families indicated significant current debt. The majority of migrant families were found to live in rural areas.

**Table 1:** **Socio-demographic characteristics and health-related factors among spouses and non-spouse caregivers of left-behind families**

**Characteristic**	**IMW spouse (n,%)**	**IMW non-spouse caregiver(n,%)**
	**N = 277**	**N = 188**
**Socio-demographic factors**		
**Gender**		
Male	119 (42.9)	8 (4.3)
Female	158 (57.0)	180 (95.7)
**Age**		
18-30	51 (18.5)	11 (5.9)
31-60	224 (81.2)	121 (64.7)
61-above	1 (0.7)	55 (29.4)
**Ethnicity**		
Sinhala	201 (72.6)	151 (81.2)
Tamil	15 (5.4)	20 (10.7)
Muslim	51 (18.4)	14 (7.5)
Other	10 (3.6)	1 (0.5)
**Education**		
No education	6 (2.2)	31 (16.6)
Primary	48 (17.3)	75 (40.1)
Secondary	223 (80.5)	81 (43.3)
**Civil status**		
Married	274 (98.9)	133 (71.1)
Unmarried	2 (0.7)	8(4.3)
Widowed/divorced	1 (0.4)	46(24.6)
**Employment**		
Non-employed	156(56.3)	141(75.4)
Employed	121(43.7)	46(24.6)
**Family debt**		
Little or no debts	153 (55.2)	102(54.3)
Significant debts	124 (44.8)	84 (44.7)
**Area of residence**		
Rural	185 (66.8)	140 (74.9)
Urban	92 (33.2)	47 (25.1)
**Health-related factors**		
**General health**		
Excellent	35 (12.6)	12 (6.4)
Fair	222 (80.1)	131 (69.7)
Poor	20 (7.2)	45 (23.9)
**Current illness**		
Current diagnosed illness	72 (26.0)	100 (53.2)
No current illness	204 (73.6)	88 (46.8)
**Healthcare visits-3 months**		
No visits	148 (53.4)	65 (34.6)
One or two visits	78 (28.2)	64 (34.0)
More than two visits	47 (17.0)	58 (30.8)

IMW: International Migrant Worker.

**Table 2:** **Migration-related factors among spouses and non-spouse caregivers of left-behind families**

**Characteristic**	**IMW spouse (n,%)**	**IMW non-spouse caregiver(n,%)**
	**N = 277**	**N = 188**
**Type of IMW employment**		
Labourer/domestic maid	164 (59.2)	106 (56.4)
Services	69 (24.9)	12 (6.4)
Technical	15 (5.4)	2 (1.1)
Professional/other	28 (10.1)	11 (5.8)
**IMW return frequency**		
Every year	53 (19.1)	10 (5.3)
Every 2-5 years	75 (27.1)	44 (23.4)
Never returned/missing	144 (52.0)	77 (40.9)
**In-bound remittance**		
Every month	155 (55.9)	53 (28.2)
Every 2-6 months/more	97 (35.0)	54 (28.7)

IMW-International Migrant Worker.

Typology of employment of the migrant worker was assessed according to the Sri Lanka Bureau of Foreign Employment (SLBFE) classification of occupations [4]. Majority (66%) belonged to the low-skilled occupation classification of ‘manual laborers’ and ‘housemaids’. Duration of employment abroad varied; 95 (23.2%) were employed for one year or less, 218 (53.2%) were employed for 1-5 years, 66 (16.1%) for 5-10 years and 29 (7.1%) were employed for over 10 years. More than half (55%) of migrant workers were reported having not returned to Sri Lanka since going abroad for work. Of those who visited, majority (29.5%) returned every two to five years. Monthly remittances in some form were received by 58% of the left-behind migrant worker families. General health was perceived to be poor by 45 (23.9%) non-spouse caregivers. Having a current diagnosed illness was reported by 100 non-spouse caregivers (53.2%). Thirty Percent of non-spouse caregivers reported of more than two visits to health care providers.

### Mental disorder prevalence

Table 3 summarizes the prevalence of CMD and constituent disorders across the groups. Overall prevalence of CMD (Depression, somatoform disorder and anxiety) was 20.7% (95%CI 16.9-24.3) in the whole sample with 14.4% (95%CI 10.3-18.6) for the spouse group and 29.8% (95%CI 23.2-36.4) for non-spouse caregiver group. Prevalence of depression was higher in the non-spouse caregiver group with 25.5% (95%CI 19.2-31.8), than in the spouse group (12.3%; 95%CI 8.3-16.1). Prevalence of Somatoform disorder in spouses was 3.6% (95%CI 1.4-5.8) and 11.7% (95%CI 7.0-16.3) in non-spouse caregivers. In the non-spouse caregiver group, 47 (25.0%) females and 1 (0.5%) male had depression. Somatoform disorder (22; 11.7%) and anxiety (7; 3.7%) was only present among the females in this group. In the spouse group, 20 (7.2%) females and 14 (5.0%) males had depression. Somatoform disorder was present among 7 (2.5%) females and 3 (1.1%) males while anxiety was present among 1 (0.4%) females and 2 (0.7%) males.

**Table 3:** **Prevalence of CMD among spouses and non-spouse caregivers of left-behind families**

**Disorder**	**Whole sample**	**IMW spouse**	**IMW family caregiver**
	**(95%CI)**	**(95%CI)**	**(95%CI)**
	**N = 410**	**N = 277**	**N = 188**
**Any CMD**	20.7 (16.9-24.3)	14.4 (10.3-18.6)	29.8 (23.2-36.4)
**Depression**	17.6 (14.1-21.1)	12.3 (8.3-16.1)	25.5 (19.2-31.8)
**Somatoform disorder**	6.9 (4.5-9.1)	3.6 (1.4-5.8)	11.7 (7.0-16.3)
**Anxiety**	2.1 (0.8-3.4)	1.1 (0.1-2.3)	3.7 (1.0-6.4)

IMW- International Migrant Worker.

### CMD associations with demographic, economic, migration-related and health-related factors

Table 4 describes the models of associations explored in the current analysis. In the unadjusted analyses, significant associations were observed between CMD and having only primary education, significant debts, in-bound remittance frequency every 2-6 months or more and poor general health for the migrant worker spouse group. Having no education, being unmarried/widowed/divorced, poor general health and 2 or more visits to a healthcare provider (during the last three months) were significantly associated with CMD in the caregiver group. Notably, having a migrant worker spouse who had not returned home since going abroad for work was significantly associated (OR 3.4; 95%CI 1.1-11.1) with CMD in the spouse group after being adjusted for age and gender. Having primary education and an in-bound remittance frequency every 2-6 months or more had slightly increased in strength after adjustment, while significant debts and poor general health showed very slight decreases in strength in the spouse group. The association between CMD and being unmarried/widowed/divorced in the caregiver group was shown to become non-significant when adjusted for age and gender. Having no education, poor general health and healthcare visits of more than 2 times (during the last three months) were still significant after adjustment, albeit decreased in strength.

**Table 4:** **Models of association between CMD and socio-economic, migration-related and health-related factors among spouse and non-spouse caregivers of left-behind families**

**Characteristic**	**IMW spouse**			**IMW non-spouse caregiver**		
	**Any CMD**	**Unadjusted**	Adjusted*	**Any CMD**	**Unadjusted**	Adjusted*
		**OR (95%CI)**	**OR (95%CI)**		**OR (95%CI)**	**OR (95%CI)**
	**N = 40**	**N = 277**	**N = 277**	**N = 56**	**N = 188**	**N = 188**
**Gender**						
Male	17 (6.1)	Reference	Reference	1 (0.5)	Reference	Reference
Female	23 (8.3)	1.0 (0.5-2.0)	1.1 (0.6-2.2)	55 (29.2)	3.1 (0.4-25.6)	3.0 (0.4- 25.2)
**Age**						
18-30	4 (1.4)	Reference	Reference	2 (1.1)	Reference	Reference
31-60	36 (13.0)	2.2 (0.8-6.6)	2.3 (0.8-6.9)	32 (17.0)	1.6 (0.3-7.9)	1.6 (0.3-7.7)
61-above	0 (0.0)	**-**	**-**	22 (11.7)	3.0 (0.6-15.2)	2.9 (0.6-14.8)
**Education**						
No education	0 (0.0)	**-**	**-**	15 (8.0)	**3.5 (1.4-8.5)**	**3.0 (1.2-7.5)**
Primary	28 (10.1)	**2.3 (1.1-5.0)**	**2.7 (1.1-6.4)**	24 (12.8)	1.8 (0.9-3.6)	1.6 (0.8-3.4)
Secondary	12 (4.3)	Reference	Reference	17 (9.0)	Reference	Reference
**Civil status**						
Married	39 (14.1)	Reference	Reference	34 (18.1)	Reference	Reference
Unmarried/widowed/divorced	1 (0.4)	**-**	**-**	22 (11.7)	**2.0 (1.0-3.9)**	1.7 (0.9-3.5)
**Employment**						
Non-employed	21 (7.6)	1.2 (0.6-2.3)	1.6 (0.6-4.2)	45 (23.9)	0.7 (0.3-1.4)	0.8 (0.4-1.8)
Employed	19 (6.8)	Reference	Reference	11 (5.8)	Reference	Reference
**Family debt**						
Little or no debts	14 (5.0)	Reference	Reference	31 (16.5)	Reference	Reference
Significant debts	26 (9.4)	**2.6 (1.3-5.3)**	**2.6 (1.3-5.2)**	24 (12.8)	0.9 (0.5-1.7)	0.9 (0.5-1.8)
**IMW return frequency**						
Every year	4 (1.4)	Reference	Reference	3 (1.6)	Reference	Reference
Every 2-5 years	10 (3.6)	1.9 (0.6-6.4)	2.1 (0.6-7.1)	11 (5.8)	0.8 (0.2-3.5)	0.9 (0.2- 4.4)
Never returned/missing	26 (9.4)	2.7 (0.9-8.1)	**3.4 (1.1-11.1)**	24 (12.8)	1.1 (0.2-4.4)	1.2 (0.3-5.6)
**In-bound remittance**						
Every month	15 (5.4)	Reference	Reference	15 (8.0)	Reference	Reference
Every 2-6 months/more	19 (6.8)	**2.3 (1.1-4.7)**	**2.7 (1.2-6.0)**	18 (9.6)	1.3 (0.6-2.9)	1.2 (0.5- 2.9)
**General health**						
Excellent/good	29 (10.5)	Reference	Reference	30 (15.9)	Reference	Reference
Poor	11 (4.0)	**9.6 (3.7-25.1)**	**9.0 (3.4-23.8)**	26 (13.8)	**5.1 (2.5-10.5)**	**4.6 (2.2-9.7)**
**Healthcare visits-3 months**						
No visits	18 (6.5)	Reference	Reference	10 (5.3)	Reference	Reference
One or two visits	13 (4.7)	1.4 (0.7-3.1)	1.5 (0.7-3.2)	16 (8.5)	1.8 (0.8-4.4)	1.6 (0.7- 4.0)
More than two visits	9 (3.2)	1.7 (0.7- 4.1)	1.6 (0.6-3.8)	30 (15.9)	**5.9 (2.5-13.8)**	**5.1 (2.2-12.1)**

IMW- International Migrant Worker *Adjusted for age and gender.

Bold values are significant at p < 0.001.

## Discussion

This is the first study in Sri Lanka to report on the mental health of adult left-behind family members (spouse and non-spouse caregivers) of IMWs. To our knowledge, this is one of the first studies exploring the broader topic in the South Asian region, measuring mental health through standardized diagnostic instruments. Some previous studies in Asia have looked at general health and psychosocial health, albeit limited to internal economic migration and elderly populations [15,20,41]. Our study, in contrast, has captured a wider representative sample of left-behind families of IMWs, highlighting both positive and negative associations at the intersection of migration and health.

### Mental disorder burden

Our findings show an increased prevalence of CMD including depression among the adult left-behind family member population, when compared to Sri Lankan national prevalence levels (CMD; 13.8%, depression; 9.1%) [35]. More importantly, the non-spouse caregiver group in the left-behind families showed more than double the burden of CMD and its constituent disorders than the spouse group. The non-spouse caregivers are older and predominantly female in according to our findings. Other studies in the Asian region have also shown high levels of depression among females and elderly left-behind family members of migrants [15,20]. However, dearth of empirical evidence on prevalence of CMD among similar populations from other countries prevent an accurate comparison and evaluation of disease burden and associated factors.

### General health and health care utilization

In the current study, the negative effects of economic migration are reflected through the high levels of CMD amongst left-behind spouses and non-spouse caregivers. The burden of providing care may come at the cost of poor mental and general health outcomes, which in turn may increase the utilization of health services. A study in Thailand linked out-migration of adult children to increased health service utilization among elderly left-behind family members, independent of socio-economic factors [15]. It is also well established that those suffering from somatoform disorders cause increased burden on health care systems [42,43]. This is especially relevant to the Sri Lankan health system, which is fully state funded and heavily subsidized.

Notably, our findings show that spouses are at increased risk of CMD when their migrant worker spouses have not returned home since going abroad. This may be due to chronic stressors linked to marital breakdown that stems from enforced separation. Together with increased burden of care for children, such enforced absences of a migrant worker spouse may lead to depression or anxiety in the left-behind spouse. However, in our study, we did not directly explore linkages between duration of separation and psychological distress. Although not directly comparable to this study on IMWs, the negative health consequences on adult members of families left-behind are similar to the study reported by Lu [2012] in an Indonesian population [20]. In addition, care-giving arrangements in left-behind families have been linked with high ‘emotional cost’ [44].

### Social, economic and migration-related factors

Most IMWs seek overseas employment with the hope of obtaining higher incomes to alleviate poverty and develop their household capital. However, at the onset of the migratory process they may incur debt. Household remittance studies have revealed the pre-migration pathways result in significant financial costs (including hidden costs due to agent exploitation) to most economic migrants and their families, especially those within low-skilled worker categories [5]. Our findings show that significant debt and decreased frequency of remittances are associated with increased CMD levels among the spouses of migrant workers, which interestingly is not observed in the non-spouse caregiver group. This may be due to the fact that spouses may have the sole responsibility in handling family finances in the absence of their wife or husband, and non-spouse caregivers may not be usually involved in managing daily economic affairs. Positive effects due to remittance sent to left-behind families may be instrumental in reducing the levels of indebtedness that prompts economic migration. Research has shown that the average wages earned by either male or female migrant workers during the first cycle of migration of two years is insufficient to cover pre-migration debts; hence the need for repeated migratory movements [45,46].

Recently, the Sri Lankan government has been promoting a more ‘skilled’ international migrant workforce. However, as yet, the vast majority of Sri Lankans entering into international labour markets are over-represented in low-skilled employment categories such as labourers and domestic maids [4,5]. Our study findings confirm this over-representation of low-skilled migrant categories, highlighting the need for a more robust action plan from the government to increase the ‘skilled’ migrant numbers.

Currently, there are no existing mechanisms to monitor the health of left-behind families of IMWs, either through public health and education systems or labour migration industry in Sri Lanka. There are no specific provisions offering mental health services to affected adult members of left-behind families. We advocate a multi-sectoral approach for monitoring the health of IMW left-behind families to be adopted at district and national levels with involvement of all relevant stake holders such as the Ministry of Health, SLBFE, provincial ministries of health, social services and public health agencies. The government and multiple stakeholders can also play a role in providing educational sessions on potential mental health issues for IMW families during SLFBE’s mandatory pre-departure training programs. We also advocate that the national migration health policy to be linked with relevant sections of national mental health policy, to enable seamless provision of mental health care for left-behind families of IMWs.

### Strengths and limitations

As the study cannot determine causality due to the cross sectional nature, prospective cohort and longitudinal studies are needed to reveal true impact of migration on physical wellbeing and mental health outcomes, and whether the workers and their families left-behind truly recover from the migration experience. The ethnic profile of the study sample closely matched national population ratios from the 2001 national population census, with 73% of migrant families being of Sinhalese ethnicity [47]. As mentioned before, the study sample represents a true cross-section of the left-behind families of migrant workers in Sri Lanka, which increases the generalisability of findings. However, the professional migrant category may be somewhat underrepresented in our sample as the SLBFE statistics does not fully cover all professional groups who migrate for work abroad. This fact and apparent sample size limitations should be considered in interpreting the associations shown. In addition, lack of a control group in our study (e.g. comparison with families without a migration history) prevents wider interpretation of findings. Whilst this study provided an insight into mental health issues faced by adult left-behind family members of migrant workers, further research is needed to explore the impact of migration on male versus female headed households, and how migration affects intra-household power dynamics and relationship outcomes.

## Conclusions

As labour migration flow increase in a rapidly developing post-conflict Sri Lanka [4,5], the impact on families left-behind leave many unanswered questions. Promoting migration for economic prosperity and ensuring health and social protection for migrants and their families remains a formidable policy challenge [48-51]. This study provides evidence on health issues among non-spouse caregivers in migrant families in Sri Lanka. Contributions from non-spouse caregivers to support the migratory process is often unrecognized by the stakeholders involved in the labour migration process and in the border discourse on migration for development. A policy process which seeks to promote the wellbeing of the left-behind families also needs to ensure ‘care for the caregiver’. The findings from this and other commissioned studies have been used to inform an evidence-based approach in formulating the National Migration Health Policy for Sri Lanka [52]. We advocate for migrant sensitive health policies as espoused within the World Health Assembly resolution (WHA 61.17), to promote migration for the benefit of all.

## References

[1] Swing WL. The State of Migration: current realities, future frontiers. Proceedings of 100^th^ IOM Council Session, 5–7 December 2011: Available: http://www.iom.int/jahia/webdav/shared/shared/mainsite/about_iom/en/council/100/MICEM_4_2011.pdf Accessed: 27 August 2012.

[2] Ghent A (2008). Overcoming Migrants’ barriers to health: bulletin of the world health organization.

[3] Central Bank of Sri Lanka. The Central Bank Annual Report 2011: Colombo: Central Bank Publications.

[4] Sri Lanka Bureau of Foreign Employment (2010). Annual statistical report of foreign employment.

[5] Economic and Social Statistics of Sri Lanka. Government of Sri Lanka, Central Bank of Sri Lanka 2012.. Available at: http://opportunitysrilanka.com/wp-content/uploads/economic-stats-2013-sl.pdf. Accessed: 27 August 2012

[6] Dissanayaka D (2003). Savings and investments of Sri Lanka returnee migrants after employment abroad. Sri Lanka J Pop Stud.

[7] UNESCAP. The high-level dialogue on International Migration and Development 2006, Available: www.unescap.org/EDC/English/Commissions/E64/E64_16E.pdf Sixty-fourth session 24-30 April 2008, Bangkok. Accessed: 27 August 2012

[8] Siriwardhana C, Wickramage K, Jayaweera K, Adikari A, Weerawarna S, Van Bortel T, et al. Impact of Economic Labour Migration: a Qualitative Exploration of Left-behind Family Member Perspectives in Sri Lanka. J Immigr Minor Health. 2014;1-10: doi:10.1007/s10903-013-9951-0 10.1007/s10903-013-9951-0 24242226

[9] Kageyama A (2008). Extent of poverty alleviation by migrant remittances in Sri Lanka. J South Asia Res.

[10] Gamburd MR (2000). The kitchen Spoon’s handle: transnationalism and Sri Lanka’s migrant housemaids.

[11] Jayaweera S, Dias M (2010). Gender roles and support networks of spouses of migrant workers. International organization for migration gender and labour migration in Asia.

[12] Hewage C, Bohlin G, Wijewardena K, Lindmark G (2010). Executive functions and child problem behaviors are sensitive to family disruption: a study of children of mothers working overseas. J Dev Sci.

[13] Athauda T, Fernando D, Nikapotha A (2000). Behavioural problems among the pre-school children of migrant mothers in Sri Lanka. J Coll Commun Phys Sri Lanka.

[14] Senaratna BCV, Perera H, Fonseka P (2011). Mental health status and risk factors for mental health problems in left-behind children of women migrant workers in Sri Lanka. Ceylon Med J.

[15] Adhikari R, Jampaklay A, Chamratrithirong A (2011). Impact of children’s migration on health and health care-seeking behavior of elderly left behind. BMC Public Health.

[16] Antman FM. The impact of migration on family left behind, discussion paper series, Forschungsinstitut zur Zukunft der Arbeit 2012, No. 6374, http://nbnresolving.de/urn:nbn:de:101:1-201206146492

[17] Gibson J, McKenzie D, Stillman S (2011). The impacts of international migration on remaining household members: Omnibus results from a migration lottery program. Rev Econ Stat.

[18] Mergo T. The effects of international migration on source households: evidence from DV lottery migration, the selected works of Teferi Mergo, 2011. available at: http://works.bepress.com/teferi_mergo/1.

[19] Antman FM (2011). The intergenerational effects of paternal migration on schooling and work: what can we learn from children’s time allocations?. J Dev Econ.

[20] Lu Y (2012). Household migration, social support, and psychosocial health: the perspective from migrant-sending areas. Soc Sci Med.

[21] Matsas R (2008). The global forum on migration and development: a new path for global governance. J Studia Diplomatica.

[22] United Nations General Assembly (2012). United Nations General Assembly Resolution on Migration and Development. Sixty-fifth Session. Resolution Item 22 (c) Adopted in 17th March 2011.

[23] Zimmerman C, Kiss L, Hossain M (2011). Migration and health: a framework for 21st century policy-making. PLoS Med.

[24] Raviola G, Becker AE, Farmer P (2011). A global scope for global health—including mental health. Lancet.

[25] Hokenstad MCT (2003). Ageing International Style: United Nations International Plan of Action on Ageing. Paper Presented to the Federation for Community Planning Human Services Institute.

[26] Wickramage K, Siriwardhana C, Siribaddana S, Vidanapathiran P, Jayasekara B, Weerawarna S, et al. Risk of mental health and nutritional problems for left-behind children of international labor migrants. BMC Psychiatry 2015, In press. 10.1186/s12888-015-0412-2 PMC4372303 25884926

[27] Lwanga SK, Lemeshow S (1991). Sample size determination in health studies.

[28] SPSS (Statistical Package for the Social Sciences Version 17.0 for Windows) (2001). SPSS for windows, Rel. 11.0.1.

[29] Spitzer RL, Williams JB, Kroenke K, Linzer M, de Gruy FV (1994). Utility of a new procedure for diagnosing mental disorders in primary care. The PRIME-MD 1000 study. JAMA.

[30] Löwe B, Kroenke K, Herzog W, Gräfe K (2004). Measuring depression outcome with a brief self-report instrument: sensitivity to change of the Patient Health Questionnaire (PHQ-9). J Affect Disord.

[31] Eack SM, Greeno CG, Lee BJ (2006). Limitations of the Patient Health Questionnaire in identifying anxiety and depression in community mental health: many cases are undetected. Res Soc Work Pract.

[32] Gilbody S, Richards D, Brealey S, Hewitt C (2007). Screening for depression in medical settings with the patient health questionnaire (PHQ): a diagnostic meta-analysis. J Gen Intern Med.

[33] PHQ Screeners. Instruction manual for PHQ and GAD-7 measures. Available at: http://phqscreeners.com/instructions/instructions.pdf Accessed on 31 January 2015

[34] Sumathipala A, Murray J (2000). New approach to translating instruments for cross‐cultural research: a combined qualitative and quantitative approach for translation and consensus generation. Int J Methods Psychiatr Res.

[35] Institute for Research and Development (IRD). National mental health survey report 2009. Colombo

[36] Siribaddana S, Ball H, Hewage S, Glozier N, Kovas Y, Dayaratne DARK (2008). Colombo Twin and Singleton Study (CoTASS): a description of a population based twin study of mental disorders in Sri Lanka. BMC Psychiatry.

[37] Siriwardhana C, Adikari A, Pannala G, Siribaddana S, Abas M, Sumathipala A (2013). Prolonged internal displacement and common mental disorders in Sri Lanka: the COMRAID study. PLoS ONE.

[38] Beecham JK, Knapp MRJ, Wing J, Thornicroft G, Brewin C (1992). Costing psychiatric interventions. Measuring mental health needs.

[39] Sumathipala A. CBT for patients with multiple complaints and/or repeated consultations. PhD Thesis 2004, University of London

[40] StataCorp (2009). Stata statistical software: release 11 2009.

[41] Abas MA, Punpuing S, Jirapramukpitak T, Guest P, Tangchonlatip K, Leese M (2009). Rural–urban migration and depression in ageing family members left behind. Br J Psychiatry.

[42] Sumathipala A (2007). What is the evidence for treatments for somatoform disorders? A critical review on previous intervention studies. Psychosom Med.

[43] Patel V, Sumathipala A (2006). Psychological approaches to somatisation in developing countries. Adv Psychiatr Treat.

[44] Ukwatta S (2010). Sri Lankan female domestic workers overseas: mothering their children from a distance. J Popul Res.

[45] Lopez-Ekra S, Aghazarm C, Kötter H, Mollard B (2011). The impact of remittances on gender roles and opportunities for children in recipient families: Research from the International Organization for Migration. Gender Dev.

[46] Ghencea B, Igor G (2004). Labour migration and remittances in the republic of Moldova.

[47] Department of Census and Statistics, Sri Lanka. Census of population and housing, 2001, Colombo, Sri Lanka.

[48] World Health Organization (2010). Health of migrants: the way forward. Report of the global consultation, Madrid, Spain.

[49] Toyota M, Yeoh B, Nguyen L (2007). Bringing the ‘left Behind’ back into view in Asia: a framework for understanding the ‘migration–left behind Nexus’. Pop Space Place.

[50] International Organization for Migration. Managing migration for the benefit of all. IOM publications 2011. Geneva, Switzerland. Available: http://www.iom.int/jahia/webdav/site/myjahiasite/shared/shared/mainsite/published_docs/books/iomfolder_eng/iom_in_brief_en.pdf]. Last accessed: 23/6/2012.

[51] Martin P. Migrants on the move in Asia. Asia-pacific issues. East West Centre, Washington Publications 1996. p. 29.

[52] Ministry of Health. National Migration Health Policy of Sri Lanka 2012, Colombo, Sri Lanka. Available at: http://www.migrationhealth.lk/sri_lanka_national_migration_health_policy.pdf Accessed on 31 January 2015.

